# A case of empyema and septic arthritis due to *Nocardia farcinica*

**DOI:** 10.1002/ccr3.1228

**Published:** 2017-10-24

**Authors:** Takashi Ishiguro, Hiroaki Yoshioka, Shoko Kawai, Shin‐ichi Katsumata, Tohru Gonoi, Noboru Takayanagi

**Affiliations:** ^1^ Department of Respiratory Medicine Saitama Cardiovascular and Respiratory Center Saitama Japan; ^2^ Department of Laboratory Saitama Cardiovascular and Respiratory Center Saitama Japan; ^3^ Department of Orthopedics Gyoda General Hospital Saitama Japan; ^4^ Medical Biology Research Center Chiba University Chiba Japan

**Keywords:** Empyema, *Nocardia farcinica*, nocardiosis, pneumoconiosis, septic arthritis

## Abstract

Septic arthritis due to *Nocardia* sp. should be suspected when a patient with risk factors such as pneumoconiosis or diabetes mellitus develops joint symptoms, especially if the patient has had nocardiosis in other sites.

## Introduction

*Nocardia* species are gram‐positive aerobic bacteria that live in soil, dust, and water. Nocardiosis is developed by inhaling these bacteria. We recently encountered a patient who developed empyema and septic arthritis (SA). To our knowledge, reports of SA due to *Nocardia* sp. are limited to sporadic cases and especially to just two cases in Japan 1, 2, and we report our case.

## Case Report

An 82‐year‐old man presented to our hospital with abnormal chest shadows (bilateral nodules and pulmonary reticular shadows) on chest X‐ray in 2000. He had a history of smoking (10–15 cigarettes daily) and alcohol intake (one glass of wine daily) but no significant family history. In April 2000, we performed bronchoscopy, but no malignant cells were detected, and no significant microorganisms were isolated. Transbronchial lung biopsy of the pulmonary nodule showed hyalinotic fibrous lesions and nodular dust containing macrophages. Many tiny needle‐like crystals were also seen in the lesion. We diagnosed him as having pneumoconiosis based on his work history (he had worked at a foundry for decades) and histologic findings of the nodules 3. The bilateral hilum nodules had been gradually increasing in size, and we continued to follow him.

**Figure 1:**
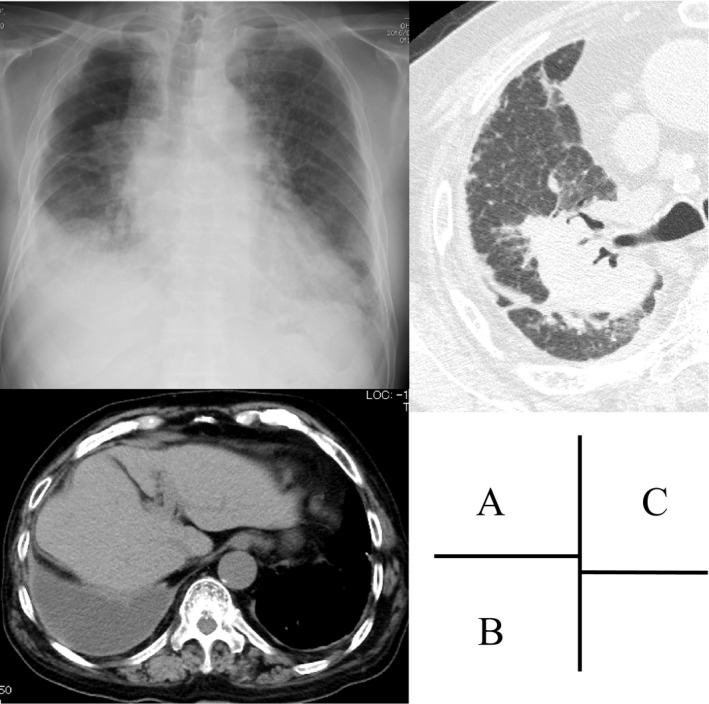
Chest imaging. Chest X‐ray showed a pulmonary nodule and right‐sided pleural effusion (A). Chest computed tomography showed pulmonary nodules, pleural effusion, and thickened pleura (B, C).

He was diagnosed as having diabetes mellitus in March 2015, and he developed a drug allergy to sulfamethoxazole‐trimethoprim (SMX‐TMP). In February 2016, the patient developed right‐sided chest pain and dyspnea on effort, and in March 2016, he developed a slight pain in his right knee joint. He presented to our hospital in the middle of March 2016. Chest X‐ray showed right‐sided pleural effusion, and chest computed tomography showed pulmonary nodules, pleural effusion, and thickened pleura (Fig. 1), whereupon he was admitted to our hospital for further evaluation.

On admission, he had a body temperature of 37.3°C, respiratory rate of 22 breaths per minute, and blood pressure of 140/72 mmHg. No anemia or jaundice and no swollen superficial lymph nodes were noted. He had not developed any heart murmurs, but his right knee was swollen, erythematous, and tender with decreased range of motion.

Laboratory data on admission showed a white blood cell (WBC) count of 8300/mm^3^ (Neu 7100/mm^3^, Eo 0/mm^3^, Baso 0/mm^3^, and Ly 900/mm^3^), Hb 14.9 g/dL, Hct 46.0%, Plt 26.4 × 10^4^/mm^3^, total protein 6.7 g/dL, BUN 16 mg/dL, Cre 0.7 mg/dL, Na 143 mmol/L, Cl 102 mmol/L, K 3.6 mmol/L, AST 25 IU/L, LDH 472 IU/L, C‐reactive protein (CRP) 10.8 mg/dL, and HbA1c 7.9%. Autoimmune antibodies were negative.

A blood culture on admission yielded no significant pathogens. Sputum culture also showed no significant pathogens including acid‐fast bacilli. Transthoracic echocardiography did not show findings suggestive of endocarditis. Thoracentesis performed on the right side on admission yielded pus (protein 1.3 g/dL, LDH 13461 IU/L, glucose 1 mg/dL, adenosine deaminase 345.2 U/mL, and crushed WBCs), and we diagnosed him as having right‐sided empyema. We inserted a chest tube and lavaged the pleural cavity with saline for several days. We administered ampicillin/sulbactam (ABPC/SBT), and his CRP level decreased. On the 2nd hospital day, a white‐colored colony developed on the blood and chocolate agar culture of the pus, and branching filamentous bacteria were isolated. *Nocardia farcinica* was identified by 16S rRNA analysis. In addition, *N. farcinica* was isolated twice from lavage saline recovered from the pleural cavity. A sensitivity test of the isolated *N. farcinica*. showed sensitivity to ampicillin/clavulanate, SMX‐TMP, minocycline (MINO), and levofloxacin (LVFX) by the disk diffusion method of the Clinical and Laboratory Standards Institute (CLSI, M24‐A). The antibiotics were changed to MINO from the 21st hospital day, and because there was no deterioration in the patient's subjective feelings, chest radiologic findings, or laboratory data, he was discharged on the 26th hospital day. His pulmonary nodules did not respond to the administered antibiotics as indicated by no change in their size or shape throughout his clinical course.

**Figure 2:**
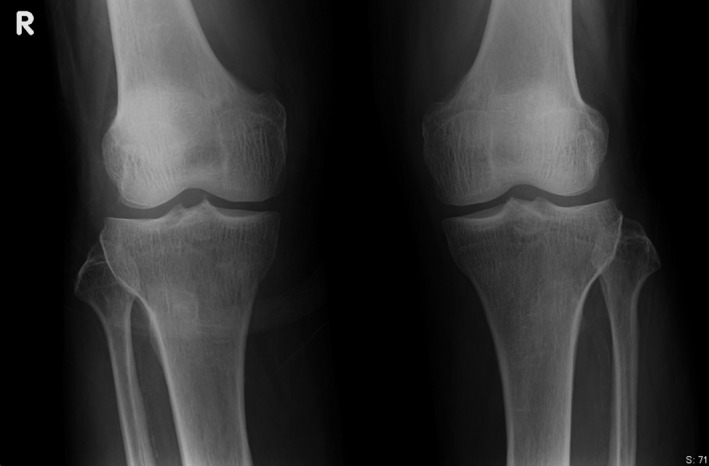
Radiograph of the knee joints. Knee radiographs showed soft tissue swelling but no bony or joint space abnormalities.

Soon after discharge, however, his knee joint pain worsened, and he was readmitted to our hospital 5 days after discharge. Laboratory data showed a WBC count of 13,100/mm^3^ and elevation of his CRP to 18.9 mg/dL. X‐rays of the right knee joint showed swelling of the joint without any abnormal bone findings (Fig. 2). Arthrocentesis of the knee joint yielded pus from which *N. farcinica* was isolated. We then diagnosed him as having SA, and a continuous drainage tube was inserted. Lavage of the joint was performed with saline, and *N. farcinica* was isolated twice from the saline recovered with the joint fluid. Drainage of the knee joint was continued for 2 weeks until negative cultures of *Nocardia* sp. from the recovered saline were verified three times. Imipenem/cilastatin was administered for 2 weeks and then was changed to LVFX. The knee swelling gradually decreased, and no fluid reaccumulated. His WBC count improved to 8800/mm^3^, and the CRP level decreased to 0.8 mg/dL, after which he was discharged in the middle of May. The LVFX was stopped in September 2016, and he has since been followed up on an outpatient basis with no relapse of the empyema or SA. He has developed no sequelae in his knee joint and has no difficulty in walking.

## Discussion

We report a patient who developed empyema and SA with underlying diseases of pneumoconiosis and diabetes mellitus. It was reported that 11 of 57 patients (19%) with pulmonary nocardiosis had pneumoconiosis 4 and that diabetes mellitus is a risk factor for nocardiosis 5. The patient improved through antibiotic therapy and drainage of the pleural cavity and right knee joint.

*Nocardia farcinica* accounts for about 10% of nocardiosis 4, 6. The present patient developed empyema, which occurs in 10–40% of the cases of nocardiosis 7, 8. However, only one of 67 reported patients with *N. farcinica* infection developed SA 9. In Japan, to our knowledge, only two other cases of SA due, respectively, to *N. nova* and *N. elegans* were reported 1, 2, but no cases of SA due to *N. farcinica* have been reported.

The infection routes of SA include blood, expansion from soft tissue surrounding joints, and direct expansion by joint operations, joint injections, or an open fracture. Our patient had not experienced injury, and the empyema preceded the SA; thus, we think hematogenous transmission likely occurred although his blood culture was negative.

The etiology of SA includes *Staphylococcus aureus* as the most frequent pathogen. It is well recognized that physicians should suspect nocardiosis when they encounter a patient who develops SA after a thorn injury 10. In addition, *Nocardia* sp. should be suspected as a cause of SA if the patient has had nocardiosis in other sites. The symptoms of our patient's knee joint at the first admission were mild, and we did not perform further evaluation of either knee joint. However, an earlier diagnosis of SA accompanying nocardiosis could have been established if we had suspected the patient of having SA because we diagnosed the patient as having empyema due to *Nocardia* sp. In a report by Chaussade et al. 11, six patients (17.6%) with SA showed some type of sequelae after treatment of the SA, but our patient experienced no sequelae.

Based on a report of 34 cases of SA due to nocardiosis 11, the median period (range) from onset to diagnosis is 20.5 (5–140) days, which indicates acute to chronic clinical onset. The median age of these patients was 50 years old, three (9%) had diabetes mellitus, as did our patient, and eight (24%) had no signs of fever. Knee joints were the most frequently involved, and hematogenous spread was found most frequently (21 patients, 70%). Furthermore, pulmonary nocardiosis was found in 17 patients (50%), but no patient had empyema. Only three patients (9%) were infected by *N. farcinica*.

We could find no abnormal radiographic findings in the knee joint of our patient other than the swollen soft tissue. Arthrocentesis revealed pus, which led to the diagnosis of SA. Useful results in detecting SA have been reported with magnetic resonance imaging 2, which might have been useful for the early diagnosis of SA in this case.

Treatment of SA includes drainage and joint lavage in addition to antibiotics. The SA in our patient worsened, and the knee joint required drainage and lavage during therapy with MINO, to which *N. farcinica* was shown to be sensitive via a sensitivity test. Early induction of drainage, lavage, and occasionally arthroscopic or direct‐vision joint synovectomy are required for SA, but in our patient, just drainage, lavage, and antibiotics administration led to improvement.

SMX‐TMP is the drug of choice for the treatment of *Nocardia* infections 12; however, our patient had a history of side effects from this drug, which are not uncommon and can cause the drug to be discontinued 6. We initially administered ABPC/SBT and subsequently changed this to MINO from the 21st hospital day. Symptoms of his knee joint worsened during the treatment with MINO, but there have been no reports of the transmission of MINO to joint fluid. Imipenem/cilastatin was administered in addition to drainage and lavage of the knee joint, and then LVFX was begun. The *N. farcinica* isolated in our patient showed sensitivity to LVFX and ciprofloxacin. Transmission of LVFX is known to be good 13, 14, and one patient with SA reported by Chaussade et al. 11 received LVFX.

We administered antibiotics to our patient for 6 months. In one textbook 12, recommendations for the period of treatment with antibiotics include (1) at least 6 months in patients with pulmonary or systemic nocardiosis (excluding brain nocardiosis) without immunosuppression, (2) at least 12 months for central nervous system nocardiosis, (3) at least 6 months for immunosuppression without HIV infection, and (4) 6–12 months for dissemination under treatment with steroids or immunosuppressant.

We report a case of nocardiosis with empyema and SA during follow‐up of a patient with pneumoconiosis. The possibility of nocardiosis should be considered when patients with risk factors such as pneumoconiosis develop empyema or SA.

## Authorship

TI: took responsibility for the integrity of the work as a whole, from inception to published article, as the guarantor of the paper. HY, Skaw, SKat, TG, and NT: aggregated the data, created the figures, and helped draft the discussion of the manuscript.

## Conflict of Interest

None declared.

## References

[1] Masaki, T. , K. Ohkusu , T. Ezaki , and H. Miyamoto . 2012 *Nocardia* elegans infection involving purulent arthritis in humans. J. Infect. Chemother. 18:386–389. 21968966 10.1007/s10156-011-0311-5

[2] Tajima, M. , A. Ishii , K. Ogawa , N. Ochiai , and H. Mishima . 2005 A case of nocardia infection of a knee joint. J. Jpn Soc. Stud. Bone Joint Infect. 19:95–97.

[3] Pneumoconiosis 2006 Pp. 127–149 *in* Katzenstein A.‐L., ed. Katzenstein and Askin's surgical pathology of non‐neoplastic lung diseases. 4th ed W. B. Saunders, Philadelphia.

[4] Kurahara, Y. , K. Tachibana , K. Tsuyuguchi , M. Akira , K. Suzuki , and S. Hayashi . 2014 Pulmonary nocardiosis: a clinical analysis of 59 cases. Respir. Investig. 52:160–166. 10.1016/j.resinv.2013.09.004 24853015

[5] Martínez Tomás, R. , R. Menéndez Villanueva , S. Reyes Calzada , M. Santos Durantez , J. M. Vallés Tarazona , M. Modesto Alapont , et al. 2007 Pulmonary nocardiosis: risk factors and outcomes. Respirology 12:394–400. 17539844 10.1111/j.1440-1843.2007.01078.x

[6] Ishiguro, T. , N. Takayanagi , T. Gonoi , M. Tamura , N. Kagiyama , S. Watanabe , et al. 2015 Clinical analysis of pulmonary nocardiosis: a retrospective, single center study. Nihon Kokyuki Gakkai‐Shi 4:133–138.

[7] Chen, Y. C. , C. H. Lee , C. C. Chien , T. L. Chao , W. C. Lin , and J. W. Liu . 2013 Pulmonary nocardiosis in southern Taiwan. J. Microbiol. Immunol. Infect. 46:441–447. 23017691 10.1016/j.jmii.2012.07.017

[8] Palmer, D. L. , R. L. Harvey , and J. K. Wheeler . 1974 Diagnostic and therapeutic considerations in *Nocardia asteroids* infections. Medicine 53:391–401. 4604319 10.1097/00005792-197409000-00005

[9] Budzik, J. M. , M. Hosseini , A. C. Jr Mackinnon , and J. B. Taxy . 2012 Disseminated Nocardia farcinica: literature review and fatal outcome in an immunocompetent patient. Surg. Infect. (Larchmt) 13:163–170. 22612440 10.1089/sur.2011.012 PMC3375863

[10] Ohl, C. A. 2010 Infectious arthritis of native joints Pp. 1443–1456 in Mandell G. L., et al., eds. Mandell, Douglas, and Bennett's principles and practice of infectious diseases, 7th ed Churchill Livingstone, Philadelphia.

[11] Chaussade, H. , D. Lebeaux , G. Gras , E. Catherinot , B. Rammaert , S. Poiree , et al. 2015 Nocardia arthritis: 3 cases and literature review. Medicine 94:e1671. 26496274 10.1097/MD.0000000000001671 PMC4620750

[12] Sorell, T. C. 2010 Nocardia species Pp. 3199–3207 in Mandell G. L., et al., eds. Mandell, Douglas, and Bennett's Principles and Practice of Infectious Diseases, 7th ed. Churchill Livingstone, Philadelphia.

[13] Asseray, N. , C. Bourigault , D. Boutoille , L. Happi , S. Touchais , S. Corvec , et al. 2016 Levofloxacin at the usual dosage to treat bone and joint infections: a cohort analysis. Int. J. Antimicrob. Agents 47:478–481. 27208901 10.1016/j.ijantimicag.2016.03.003

[14] Izumi, T. , M. Itoman , and M. Sato . 1993 Study on the transfer of levofloxacin to join fluid or synovium. Igaku To Yakugaku 42:993–996.

